# An architecture for building cohorts of images from real-world clinical data from the whole Scottish population supporting research and AI development.

**DOI:** 10.23889/ijpds.v7i3.1916

**Published:** 2022-08-25

**Authors:** Emily Jefferson, Susan Krueger, Ruairidh Macleod, James Sutherland, Thomas Nind, Roy Mudie, Bianca Prodan, Andrew Brooks, Robert Wallace, Carole Morris, Jacqueline Caldwell, Rob Baxter, Mark Parsons

**Affiliations:** 1 University of Dundee; 2 University of Edinburgh; 3 Public Health Scotland

## Objectives

To research and develop tools and methods for building cohorts of images linked to longitudinal healthcare records from real-world clinical images from the whole Scottish population. To provide this capability for the Scottish Medical Imaging service (provided by the Scottish National Safe Haven) to support research and AI projects.

## Approach

Clinical images, especially when linked to routinely collected health data, are extremely useful for many types of research and AI development. However, finding and using clinical images for research data is challenging because:

1. Existing software used to search for images are designed for clinical care rather than research making it easy to find images for a particular patient. They are not designed to search for all images with particular characteristics e.g. slice thickness/scanning protocol/contrast agent/patient medication.
2. Reuse of clinical images for research requires de-identification, yet identifiable data can be present in many areas of the associated image file.

## Results

The PICTURES (InterdisciPlInary Collaboration for efficienT and effective Use of clinical images in big data health care RESearch) 5-year programme has developed an architecture for building cohorts of images based upon research criteria and providing these in a di-identifiable form within a Safe Haven environment. There are 3 zones:

- An identifiable zone which stores the raw image data and a MongoDB database which captures the metadata
- A de-identified zone which provides a database and tools for cohort building which do not require imaging data expertise
- Several Project Private Zones (PPZs) where researchers can install custom software and access the de-identified images for their project

The architecture supports cohort building based upon features within pixel data, image metadata and linking to longitudinal health care records.

## Conclusion

PICTURES is currently enhancing the cohort building user interface used by the National Safe Haven and supporting exemplar projects. The SMI service is live and accepting requests for more information. The software is open source and we welcome the use of the platform by other Safe Havens/research groups.

